# Custom barcoded primers for influenza A nanopore sequencing: enhanced performance with reduced preparation time

**DOI:** 10.3389/fcimb.2025.1545032

**Published:** 2025-04-15

**Authors:** Iryna V. Goraichuk, David L. Suarez

**Affiliations:** Southeast Poultry Research Laboratory, U.S. National Poultry Research Center, Agriculture Research Service, U.S Department of Agriculture, Athens, GA, United States

**Keywords:** next-generation sequencing, NGS, WGS, influenza, nanopore, MinION, flongle

## Abstract

Highly pathogenic avian influenza is endemic and widespread in wild birds and is causing major outbreaks in poultry worldwide and in U.S. dairy cows, with several recent human cases, highlighting the need for reliable and rapid sequencing to track mutations that may facilitate viral replication in different hosts. SNP analysis is a useful molecular epidemiology tool to track outbreaks, but it requires accurate whole-genome sequencing (WGS) with sufficient read depth across all eight segments. In outbreak situations, where timely data is critical for controlling the spread of the virus, reducing sequencing preparation time while maintaining high-quality standards is particularly important. In this study, we optimized a custom barcoded primer strategy for influenza A whole-genome sequencing on the nanopore sequencing platform, combining the high performance of the Native Barcoding Kit with the prompt preparation time of the Rapid Barcoding Kit. Custom barcoded primers were designed to perform barcode attachment during RT-PCR amplification, eliminating the need for separate barcoding and clean-up steps, thus reducing library preparation time. We compared the performance of the custom barcoded primer method with the Native and Rapid barcoding kits in terms of read quality, read depth, and sequencing output. The results show that the custom barcoded primers provided performance comparable to the Native Barcoding Kit while reducing library preparation time by 2.3X compared to the Native kit and being only 15 minutes longer than the Rapid kit with better depth of sequencing. Additionally, the custom barcoded primer method was evaluated on a variety of clinical sample types. This approach offers a promising solution for influenza A sequencing, providing both high throughput and time efficiency, which significantly improves the time-to-result turnaround, making sequencing more accessible for real-time surveillance.

## 1 Introduction

Influenza A virus, a segmented RNA virus in the *Orthomyxyoviridae* family and *Alphainfluenzavirus* genus, is a highly variable and widespread pathogen responsible for seasonal flu outbreaks in humans, as well as significant diseases in animals (Suarez, 2017; Swayne et al., 2020; WHO, 2023). It can infect a wide range of hosts, including birds, mammals, and humans, and is known for its rapid genetic changes, which can lead to alterations in transmissibility, virulence, and host range. These genetic changes are primarily driven by antigenic drift, which involves the accumulation of point mutations, and antigenic shift, which occurs through reassortment of gene segments during co-infection with different influenza A viruses. Both processes enable the virus to adapt to different hosts and environmental conditions (García et al., 1997; Manrubia et al., 2005; Reperant et al., 2009; Kandeil et al., 2023; Graziosi et al., 2024). Recently, the H5N1 highly pathogenic avian influenza A virus has become a major concern. In the current 2020-2024 panzootic, H5N1 detection was reported in over 48 mammal species (Plaza et al., 2024), underscoring the growing threat of cross-species transmission. A notable example of this is the ongoing outbreaks of H5N1 avian influenza in U.S. dairy cows, which have affected over 500 dairy herds and resulted in more than 50 human cases (Caserta et al., 2024; CDC, 2024; Spackman et al., 2024; Uyeki et al., 2024; Suarez et al., 2025). This highlights the serious risk of avian influenza adapting to non-avian species, including mammals, which could lead to further public health and economic challenges (Barbachano-Guerrero et al., 2023; Kang et al., 2024; Koopmans et al., 2024). Whole-genome sequencing (WGS) of influenza A viruses is an essential tool for identifying mutations associated with viral evolution, transmission, and pathogenicity (Dinis et al., 2016; Suttie et al., 2019; Sun et al., 2020; Leyson et al., 2023; Youk et al., 2023; de Carvalho Araujo et al., 2024; Guo et al., 2024; Kim et al., 2024; Lee et al., 2024; Powell et al., 2024). However, in outbreak situations, rapid data acquisition is critical to controlling the spread of the virus, necessitating efficient sequencing methods with reduced library preparation time but without sacrificing high-quality standards. Thus, there is an urgent need for reliable and rapid sequencing methods for the surveillance of influenza A viruses, particularly in panzootic situations like H5N1, where timely interventions and control measures are crucial.

Targeted sequencing is particularly advantageous, as it focuses on amplifying specific regions of the genome, ensuring high efficiency and accuracy. Influenza A has conserved termini present at both the 3’- and 5’-ends of each genome segment, which enable the use of universal primers for influenza whole-genome amplification, commonly used for further amplicon sequencing (Wang et al., 2015; Hoffmann et al., 2001; Zhou et al., 2009; Imai et al., 2018; Mitchell et al., 2021; Chauhan and Gordon, 2022; Ip et al., 2023). In our previous work, we optimized an RT-PCR protocol for influenza whole-genome amplification, specifically addressing improvements to enhance subsequent sequencing outcomes (Goraichuk et al., 2024a). This optimization increases the specificity of the sequencing, reduces the amplification of non-target sequences, and ensures uniform coverage of all eight viral segments, which is critical for accurate genomic analysis. The optimized protocol enables influenza whole-genome amplification with high coverage, making the amplification products suitable for sequencing on various sequencing platforms.

Among different sequencing platforms, Oxford Nanopore Technologies (ONT) platforms, such as the MinION, offer significant advantages, particularly in their near real-time data generation and long-read capabilities (Pugh, 2023). Nanopore sequencing works by passing nucleic acids through a nanopore, and the resulting electrical signal is translated into sequence data. This method allows for stopping the sequencing run once sufficient data is acquired, making it especially valuable in outbreak scenarios where quick results are needed. The ability to sequence long reads is particularly useful for improving genome assembly and providing more accurate results for complex genomes (Warburton and Sebra, 2023; Hall et al., 2024). It is also advantageous for sequencing long amplicons without the need for prior fragmentation, which is particularly useful for detecting recombination events. Additionally, the MinION provides portability, enabling sequencing in different environments, from centralized labs to field settings (Faria et al., 2016; Hoenen et al., 2016; Karamendin et al., 2016; McIntyre et al., 2016; Quick et al., 2016; Castro-Wallace et al., 2017; Johnson et al., 2017; Parker et al., 2017; Pomerantz et al., 2018; Gowers et al., 2019; Carr et al., 2020; Urban et al., 2021).

Combining the ability to simultaneously amplify all influenza genome segments in one RT-PCR reaction with the advantages of nanopore sequencing provides a reliable and rapid sequencing approach that has been successfully used for the surveillance of influenza A viruses (King et al., 2022; Miah et al., 2023; Plancarte et al., 2023; Baybay et al., 2024; Lagan et al., 2024; Maqsood et al., 2024; Siegers et al., 2024; Wang et al., 2024). ONT offers the Ligation Sequencing Influenza Whole Genome V14 protocol, which utilizes the Native Barcoding Kit for sample multiplexing by adding unique barcodes to each sample (Oxford_Nanopore_Technologies, 2024a). This enables the pooling of multiple samples into a single sequencing run, increasing efficiency and reducing costs. There are two ONT barcoding kits available – the Native and Rapid Barcoding Kits – which use different chemistries for barcode and adapter attachment (Oxford_Nanopore_Technologies, 2024b). The Native Barcoding Kit employs ligation-based chemistry, optimized for accuracy and high read output. In contrast, the Rapid Barcoding Kit uses transposase-based chemistry for faster barcode attachment and fragmentation, followed by rapid-based adapter attachment for quicker library preparation. However, this results in reduced read lengths and sequencing output. While the Rapid kit’s faster preparation time makes it ideal for situations requiring quick turnaround, the Native kit provides more reliable, higher-quality data, making it particularly advantageous for comprehensive genomic analysis.

To address the need for both speed and high-quality data, this study aims to optimize influenza A whole-genome sequencing on the nanopore sequencing platform by developing a custom barcoded primer strategy. This strategy enables barcode attachment during RT-PCR amplification, eliminating the need for separate barcoding and clean-up steps. As a result, it reduces library preparation time, bringing it closer to the efficiency of the Rapid Barcoding Kit while maintaining the high output and accuracy of the Native Barcoding Kit through ligase-based adapter attachment. We compare the performance of this custom barcoded primer method with the Native and Rapid barcoding kits in terms of read quality, read depth, and overall sequencing output. This optimized method aims to provide a balanced solution for influenza A sequencing, offering both time efficiency and high-quality results, making it suitable for surveillance and outbreak management.

## 2 Materials and methods

### 2.1 Samples

Eight avian influenza isolates of varying virulence and subtypes (**Table 1**) from the Southeast Poultry Research Laboratory (SEPRL) repository were propagated in 9–11-day-old specific-pathogen-free (SPF) embryonated chicken eggs (Senne, 2008). The allantoic fluids harvested from these eggs were used to compare different barcoding strategies for nanopore sequencing in this study. Background information on the egg-grown isolates, including details on their host, country of origin, year of collection, pathogenicity, subtype, and GenBank accession numbers, is summarized in **Table 1**.

**Table 1:** Background information on influenza A viruses used for the comparison of barcoding methods in this study.

Isolate ID	Host	Country	Year of collection	Pathogenicity	Subtype	RT-qPCR, Ct ^3^	GenBank accession no.
F12505B	Chicken	Egypt	2016	HPAIV ^1^	H5N1	17.6	PQ064247 - PQ064254
MX/37905	Chicken	Mexico	2015	HPAIV	H7N3	11.6	PQ106540 - PQ106540, MH342039
NSW/3121-1	Chicken	Australia	2012	HPAIV	H7N7	15.2	PQ064551 - PQ064558
1158-11406-1	Chicken	England	2008	HPAIV	H7N7	11.2	PQ064115 - PQ064122
PA/35154	Chicken	USA	1991	LPAIV ^2^	H1N1	12.4	EU735794 - EU735801
TX/G021090002	Chicken	USA	2002	LPAIV	H5N3	16.7	PQ064267 - PQ064274
CA/K0301417	Chicken	USA	2003	LPAIV	H6N2	11.6	PQ064136 - PQ064143
CO/169118-13	Turkey	USA	2002	LPAIV	H8N4	12.5	GU051913 - GU051917, PQ060363 - PQ060365

^1^Highly pathogenic avian influenza virus; ^2^Low pathogenic avian influenza virus; ^3^Cycle threshold.

The developed PCR method utilizing barcoded primers was further tested on various clinical samples, which included bovine mammary gland tissues, cat brain tissues, and chicken brain, muscle, heart, spleen, oropharyngeal (OP), and cloacal (CL) samples (**Table 2**). The chicken samples were collected from SPF chickens infected with different avian influenza virus isolates. Background information on the clinical samples, including host, sample type, subtype, and GISAID accession numbers (Khare et al., 2021), is summarized in **Table 2**.

**Table 2:** Background information on clinical samples used in this study.

Sample No.	Sample Type	Host	Isolate Name	Subtype	RT-qPCR, Ct ^3^	GISAID accession no.
1	Mammary gland	Dairy cow	A/dairy cow/Texas/A240750066-18/2024	H5N1	17.9	EPI_ISL_19334243
2	Mammary gland	Dairy cow	A/dairy cow/Texas/A240750066-18/2024	H5N1	16.5	EPI_ISL_19334243
3	Brain	Cat	A/cat/New Mexico/F001/2024	H5N1	8.8	EPI_ISL_19696071
4	Muscle	Chicken	A/American wigeon/South Carolina/22-000345-001/2021	H5N1	19.6	EPI_ISL_18133029
5	Muscle	Chicken	A/bald eagle/Florida/22-006544-004-original/2022	H5N1	18.3	EPI_ISL_18132941
6	Heart	Chicken	A/chicken/Idaho/22-011347-004-original/2022	H5N1	20.1	EPI_ISL_15077371
7	Spleen	Chicken	A/skunk/Washington/22-019274-001-original/2022	H5N1	19.7	EPI_ISL_15078254
8	Brain	Chicken	A/skunk/Washington/22-019274-001-original/2022	H5N1	18.0	EPI_ISL_15078254
9	Heart	Chicken	A/skunk/Washington/22-019274-001-original/2022	H5N1	15.2	EPI_ISL_15078254
10	OP ^1^	Chicken	A/turkey/Indiana/22-003707-003/2022	H5N1	21.6	EPI_ISL_9909371
11	CL ^2^	Chicken	A/turkey/Indiana/22-003707-003/2022	H5N1	21.6	EPI_ISL_9909371
12	OP	Chicken	A/turkey/Minnesota/15-012582-1/2015	H5N2	23.2	EPI_ISL_225571

^1^Oropharyngeal swab sample; ^2^Cloacal swab sample; ^3^Cycle threshold.

### 2.2 RNA extraction and RT-qPCR

Total RNA was extracted from infectious allantoic fluids and clinical samples using the MagMAX™-96 AI/ND Viral RNA Isolation Kit (Applied Biosystems, USA) following the manufacturer’s instructions. RNA quality and concentrations were assessed using the EzDrop 1000C spectrophotometer (Blue-Ray Biotech, Taiwan). The presence of influenza RNA was confirmed using the avian influenza matrix gene RT-qPCR assay, as previously described (Spackman et al., 2002; Goraichuk et al., 2024). Extracted viral RNA were then used for the comparison of different ONT barcoding strategies.

### 2.3 Nanopore library preparation and sequencing

To compare the Native, Rapid, and PCR barcoding strategies, three nanopore sequencing libraries were prepared using the Native Barcoding Kit 24 V14 (SQK-NBD114.24), Rapid Barcoding Kit 24 V14 (SQK-RBK114.24), and Ligation Sequencing Kit V14 (SQK-LSK114), respectively. The resulting datasets were designated as Native, Rapid, and PCR libraries, corresponding to the respective barcoding processes used in each library preparation strategy. For both the Native and Rapid barcoding methods, amplicons were generated using multisegment RT-PCR amplification to simultaneously amplify all influenza A genome segments from 8 isolates. This was done following our previously described method using Opti primers in conjunction with the LunaScript^®^ Multiplex One-Step RT-PCR Kit, New England Biolabs, USA) (Goraichuk et al., 2024a; Goraichuk et al., 2024b).

It is important to note that Rapid chemistry is known to be unable to capture the entire amplification product due to transposase activity during barcode attachment, which results in 15-20 nt being truncated at both termini. Therefore, the ONT protocol recommends designing primers that include an extra 15-20 bp at the start and end of the actual target sequence. The Opti primers used in this study follow this guidance and, in addition to the conserved influenza termini, include a 24-nucleotide overhanging tail to ensure complete target coverage.

To reduce preparation time while maintaining the high output and accuracy of ligation-based chemistry, we designed custom barcoded primers to incorporate barcoding during RT-PCR amplification with subsequent ligation of the sequencing adapters. These barcoded primers were designed similarly to Opti primers used in the Native and Rapid runs, consisting of two parts: the conserved influenza termini sequences and an overhanging tail. The key difference between the Opti and barcoded primers is that, in the barcoded primers, the overhanging tail corresponds to ONT’s barcode sequence. Thereby, each barcoded primer included the corresponding barcode sequence from the PCR Barcoding Expansion (EXP-PBC096, Oxford Nanopore Technologies, England) at the 5’-end, followed by the influenza Uni 12 and Uni 13 conserved termini at the 3’-end (**Supplementary Table 1**). Custom barcoded primer sets were purchased from Integrated DNA Technologies (Coralville, USA). For the PCR barcoding method, amplicons were generated using the LunaScript^®^ Multiplex One-Step RT-PCR Kit, New England Biolabs, USA) with the same cycling conditions as those used for the Native and Rapid barcoding methods (Goraichuk et al., 2024a). Briefly, 50-µL reaction volumes comprised of 5 µL of total RNA, 10 µL of LunaScript Multiplex One-Step RT-PCR Reaction Mix (5X), 2.5 µL of 20 µM working primer mix solution, 2 µL of LunaScript Multiplex One-Step RT-PCR Enzyme Mix (25X), and 30.5 µl of sterile nuclease-free water. Working primer solution per each barcode was prepared by combining 100 µM stock solutions of F1, F2, and R primers from barcoded primer set in 0.35:0.65:1 ratio. RT-PCR amplification process included an initial RT step (90 min at 55°C), RT inactivation/initial denaturation (1 min at 98°C), and PCR steps of 5 cycles (10 s at 98°C, 30 s at 44°C, and 3 min 30 s at 72°C) and 30 cycles (10 s at 98°C, 30 s at 69°C, and 3 min 30 s at 72°C), followed by final extension (10 min at 72°C). Detailed protocol have been deposited at protocols.io: dx.doi.org/10.17504/protocols.io.5qpvo93e7v4o/v1 (Goraichuk and Suarez, 2024).

All amplicons were purified using the Select-a-Size DNA Clean & Concentrator (Zymo Research, USA), which has previously been shown to reduce purification time and increase efficiency in removing short reads, resulting in more uniform coverage across the polymerase segments (Goraichuk et al., 2024b). After purification, 200 fmol of amplicons were barcoded and adapters were attached using ligation-based chemistry for Native barcoding (Oxford_Nanopore_Technologies, 2024c) and rapid-based chemistry for Rapid Barcoding Kit (Oxford_Nanopore_Technologies, 2024d), in accordance with the manufacturer’s recommendations. For PCR barcoding, 200 fmol total (25 fmol per sample) of barcoded amplicons were used for further adapter ligation using Ligation Sequencing Kit V14 (Oxford_Nanopore_Technologie, 2024). Molarity was calculated based on an assumed average size of 2,000 bp for multi-segment RT-PCR fragments. The Native library preparation requires additional third-party consumables, including Blunt/TA Repair Mix, Ultra II End repair/dA-tailing Module, and Quick Ligation Module. The Rapid library preparation, in contrast, does not require any of these consumables. The PCR method, using custom barcoded primers, eliminates the need for Blunt/TA Ligase Master Mix but still requires the Ultra II End repair/dA-tailing Module and Quick Ligation Module. This difference in required consumables is the primary driver of the cost difference between the Native and Rapid methods.

The final libraries for each of the Native, Rapid, and PCR barcoding strategies were quantified using the High Sensitivity D5000 Screen Tape on a 4150 TapeStation (Agilent Technologies, USA) and Qubit 1X dsDNA High Sensitivity Kit on a Qubit 4 fluorometer (Invitrogen, USA). We then prepared 15 fmol of Native and PCR libraries in 5 µl, and 5.5 µl of the Rapid final prepared library, which were loaded onto separate R10.4.1 Flongle flow cells (FLO-FLG114, Oxford Nanopore Technologies, England) for sequencing using the Mk1C sequencer with MinKNOW 23.04.8 software. Each sequencing run lasted for 6 hours. Additionally, the PCR method was tested on clinical samples by sequencing 50 fmol for 6 hours on a MinION flow cell (FLO-MIN114, Oxford Nanopore Technologies, England) using the Mk1C with MinKNOW 24.11.8 software.

### 2.4 Bioinformatics analysis

The nanopore raw Pod5 files from all runs were basecalled using the MinKNOW 23.07.12 (bionic) software on a MinION Mk1C instrument with a high-accuracy algorithm to generate FastQ files. Basecalled reads with a minimum Q-score of 9 and a minimum length of 200 bp were classified as “PASS”. The Native and Rapid runs were then demultiplexed and trimmed using Dorado basecaller server 7.3.11 within the MinKNOW, while basecalled “PASS” reads from the PCR barcoding run were demultiplexed using Dorado 0.7.3 with settings for the custom barcoded primers (**Supplementary Table 1**). Nextflow workflow for demultiplexing libraries prepared with the custom barcoded primers can be found at https://github.com/Goraichuk/Dorado_FluA_Custom_Demultiplexing. All demultiplexed reads were further analyzed on the Galaxy platform (The Galaxy Community, 2024). Run statistics were generated using NanoPlot (De Coster et al., 2018) and NanoporeQC (Lanfear et al., 2019). Influenza genomes were assembled by aligning filtered reads to concatenated reference segments of influenza viruses previously sequenced using Illumina technology, with alignment performed using minimap2 (Li, 2018) and verified in Geneious Prime 2023.0.1. To mitigate potential bias from transposase-mediated truncation of termini during rapid-based barcode attachment, sequencing runs were re-analyzed using shorter reference sequences containing only coding regions for reference mapping assembly. Final consensus sequences were generated using the bam2consensus tool (Volkening, 2023) and assembly polishing tool Medaka. The coverage of the influenza virus genome was determined using SAMtools depth (Andrés et al., 2023).

### 2.5 Statistical analysis

GraphPad Prism 10.2.3 (Sović et al., 2016) was used for data visualization and statistical analysis. A one-way ANOVA followed by Tukey’s multiple comparisons test was employed to compare the relative differences in the average number of mapped reads, mean read depth, and minimum read depth coverage per sample among Native, Rapid, and PCR runs, with eight influenza A viruses sequenced in each run. The *p*-value ≤ 0.05 was considered statistically significant.

## 3 Results

### 3.1 Barcoding efficiency

In our efforts to optimize library preparation for high output, high accuracy, and a quick preparation time for the influenza A whole-genome nanopore sequencing, we designed and tested barcoded primers to perform barcoding during RT-PCR amplification (Goraichuk and Suarez, 2024), followed by ligase-based adapter attachment. After testing various combinations (data not shown), we determined that the presence of the barcode sequence at both the forward and reverse primers increased demultiplexing rate compared to barcoded only forward or reverse primer. Determined optimal custom barcode arrangements and corresponding Dorado demultiplexing settings are provided in **Supplementary Table 1**. This approach aims to maintain the higher output and accuracy of the ligase-based Native Barcoding Kit while reducing preparation time to align more closely with the throughput of the rapid-based Rapid Barcoding Kit. To verify this, the performance of the PCR barcoding method was compared to both the Native and Rapid barcoding kits.

The Native and PCR runs, both utilizing ligation-based chemistry of adapter attachment, yielded a significantly higher number of raw reads – 231,480 and 226,079, respectively (**Table 3**), while the Rapid run, as expected, produced fewer reads – 183,860, resulting in a 1.2X reduction compared to the ligase-based methods. Over 80% of raw reads were successfully basecalled in all three runs, with the highest percentage (84.4%) of basecalled reads produced by the PCR method. As expected, the Rapid run generated shorter reads due to the rapid-based chemistry, which uses the transposase method for barcode attachment, leading to fragmentation of amplicons.

**Table 3:** Summary statistics of nanopore sequencing runs of NGS libraries prepared with Native, Rapid, and PCR barcoding strategies.

Barcoding Method	Total Yield, Gb	Raw Reads	Basecalled Reads (Q > 9), %	Demultiplexed Reads, %	Unclassified Reads, %	Incorrect Barcodes/Reads	Mean Read Length, bp	Mean Quality
Native	0.28	231,480	81.0	97.2	2.8	2/2	1207.7	13.1
Rapid	0.13	183,857	82.7	87.3	12.6	9/30	730.6	12.9
PCR	0.28	226,079	84.4	92.0	8.0	0/0	1248.1	12.5

**Figure 1:**
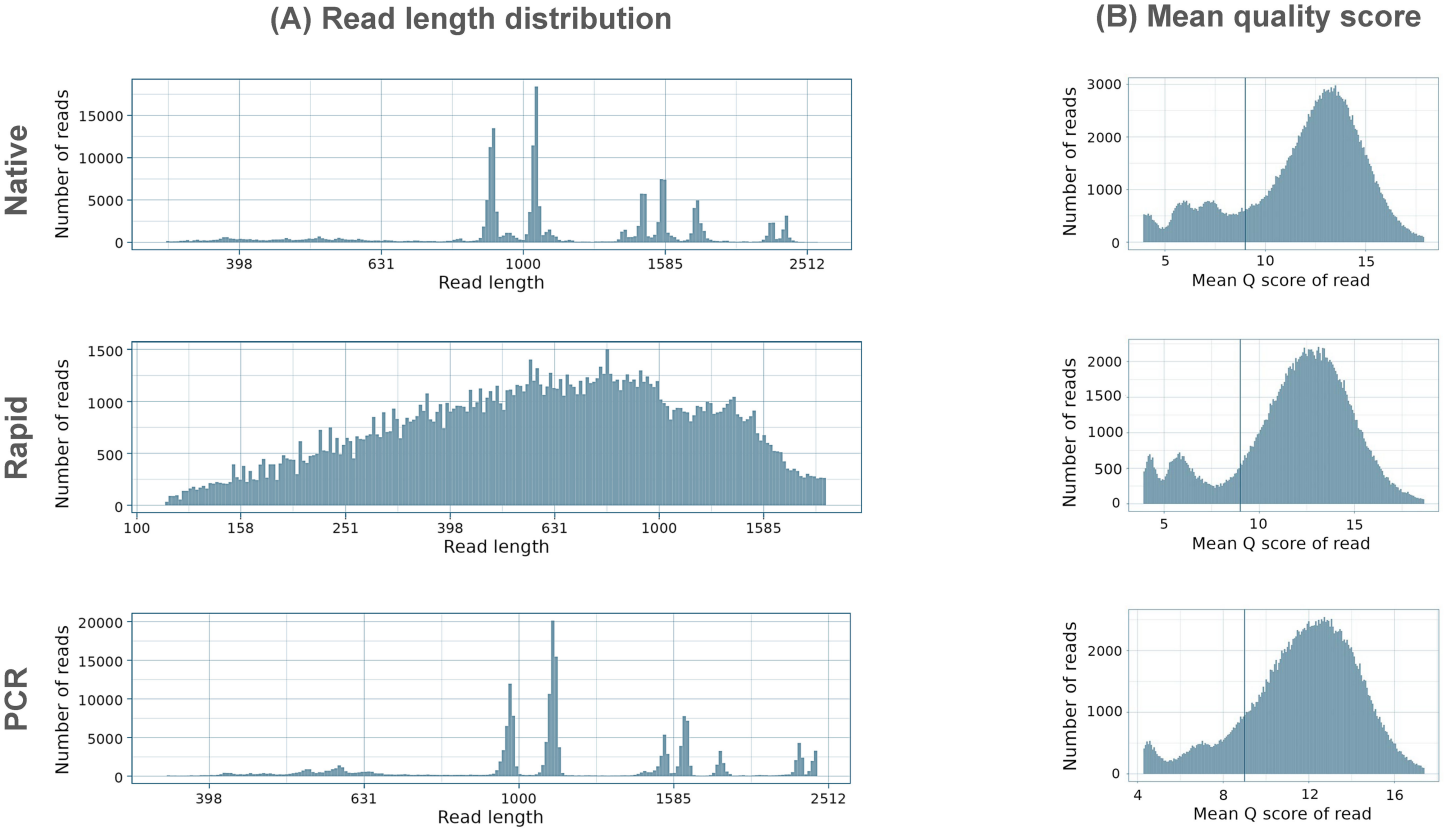
Summary of **(A)** read length distribution and **(B)** mean quality distributions in Native, Rapid, and PCR sequencing runs. The y-axes are scaled differently for each run to ensure a clear visualization of the distribution characteristics of each dataset.

The PCR run provided the highest mean read length of 1248.1 bp, closely followed by the Native run with a mean length of 1207.7 bp, while the Rapid run, as expected, produced shorter reads with a mean length of 730.6 bp (**Figure 1A**) due to the rapid-based chemistry, which uses the transposase method for barcode attachment, leading to fragmentation of amplicons (**Figure 1A**). The majority of reads in the Native and PCR runs were distributed in accordance with the length of the eight influenza genome segments, while reads in the Rapid run were fragmented into different sizes. In all three runs, the majority of reads exceeded the minimum accepted Q9 quality threshold, with the mean quality scores of demultiplexed reads ranging between 12.5 and 13.1, the highest being achieved in the Native run and the lowest in the PCR run (**Figure 1B**).

**Figure 2:**
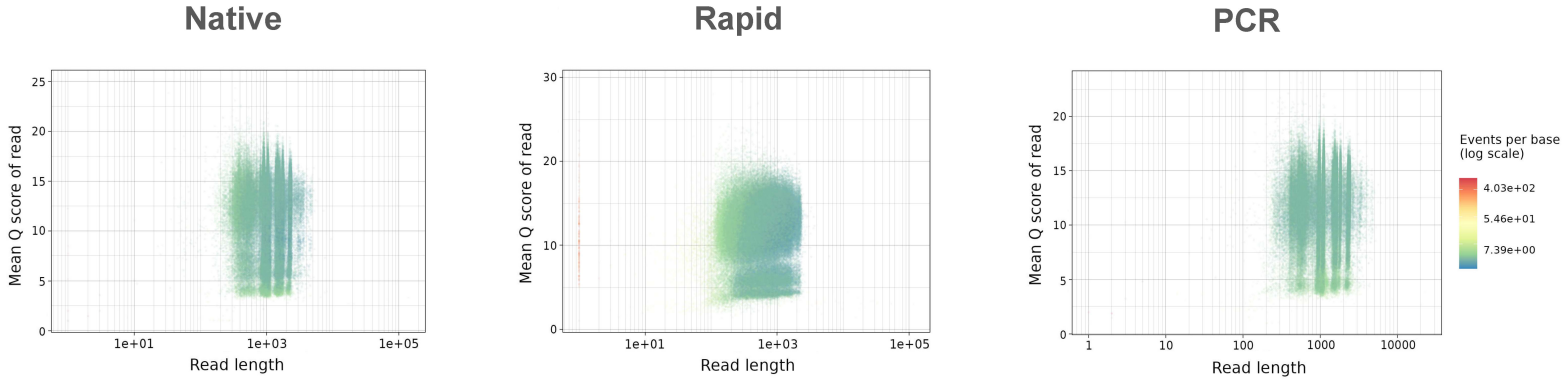
Summary of read length vs quality distribution in Native, Rapid, and PCR sequencing runs.

The quality distribution of all sequenced reads was similar in both the Native and PCR runs, with patterns closely matching the length distribution of eight influenza genome segments, (**Figure 2**). In these runs, the majority of reads exhibited consistent quality across varying read lengths, following the typical distribution of influenza genome segments. In contrast, the Rapid run generated a large number of reads with a notable presence of shorter reads with varying quality.

The Native run with the ligase-based barcoding method resulted in the highest percentage (97.2%) of demultiplexed reads out of basecalled, followed by the PCR method with barcoded primers (92.0%). The Rapid run, utilizing rapid-based barcode attachment chemistry, showed the lowest rate of demultiplexing (87.3%), indicating less efficient barcode attachment compared to the other two methods (**Table 3**). This inefficiency in demultiplexing affected the percentage of unclassified reads, with the Rapid run exhibiting the highest unclassified read rate at 12.6%. Additionally, both ligase- and rapid-based barcode attachments led to incorrect barcode assignment. Specifically, 2 incorrect barcodes were assigned in the Native run, and 9 incorrect barcodes were assigned in the Rapid run. However, the number of incorrectly barcoded reads was very low in both cases. The Dorado settings for custom demultiplexing of barcoded primers in the PCR run were optimized to minimize incorrect barcode assignment, resulting in no incorrect barcodes being assigned. The optimized setting can be found in **Supplementary Table 1**.

### 3.2 Ligase-based chemistry outperformed rapid-based chemistry

**Figure 3:**
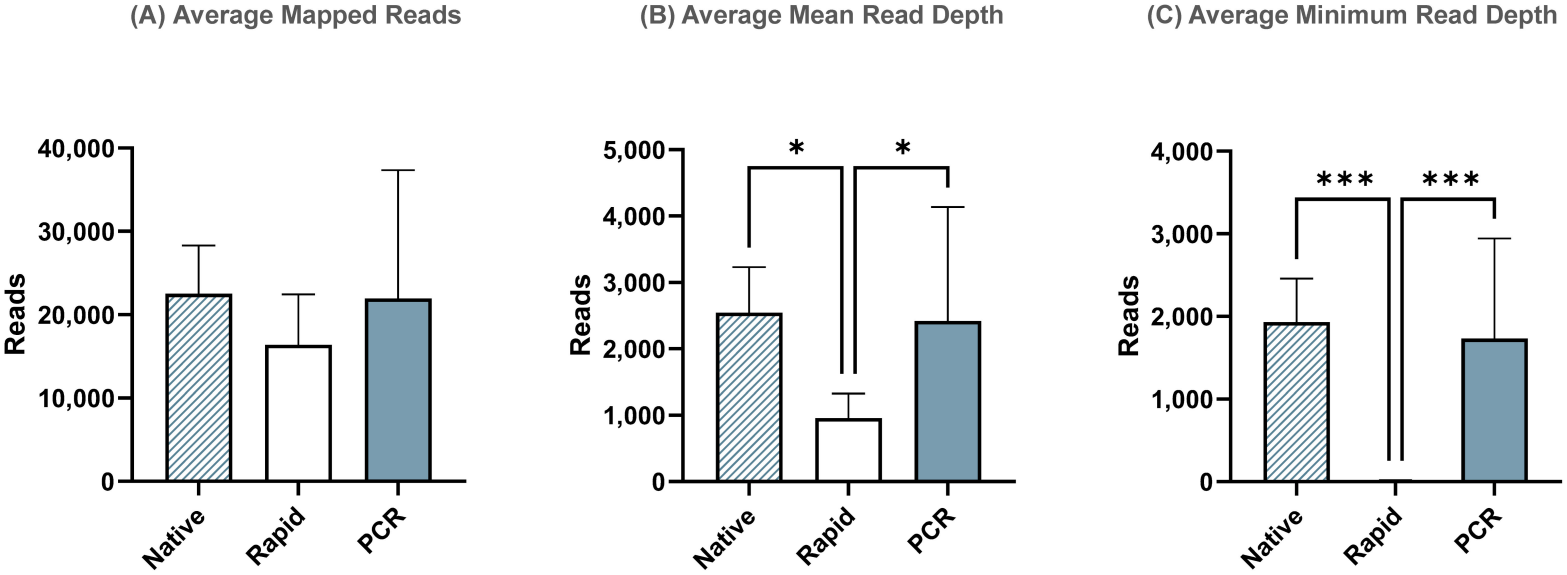
Sequencing summary for comparison of Native, Rapid, and PCR barcoding strategies’ performance with data mapped to full-length reference genomes. Average mapped reads **(A)**, mean read depth **(B)**, and minimum read depth **(C)** across all segments of eight different influenza A viruses. *P*-value is defined as follows: *p ≤ 0.05, ***p ≤ 0.001.

After reference mapping assembly of influenza complete genomes, we identified that the PCR run yielded the highest percentage (99.97%) of influenza reads out of all demultiplexed reads, while the Native and Rapid run provided 98.8% and 98.7%, respectively (**Table 4**). Although the difference in number of average reads mapped across the influenza genome was not statistically significant between all three runs (**Figure 3A**), the average mean and minimum depth of mapped reads produced in the Rapid run were significantly lower compared to both ligase-based Native and PCR runs (**Figures 3B, C**). This indicates that even though the expected reduction in total influenza read numbers was not significant between all runs, the ligase-based chemistry outperformed the rapid-based chemistry in providing better read depth, which, in turn, resulted in higher genome coverage. Both of these factors are essential for reliable SNP analysis.

**Table 4:** Summary statistics of influenza A genome assembly using Native, Rapid, and PCR barcoding strategies for NGS library preparation.

Barcoding Method	Complete Genome				Coding Sequence			
	Total Influenza Reads	Average Mapped Reads	Average Mean Read Depth	Average Min Read Depth	Total Influenza Reads	Average Mapped Reads	Average Mean Read Depth	Average Min Read Depth
Native	180,215	22,527	2,544	1,933	180,201	22,525	2,540	1,860
Rapid	130,985	16,373	957	14	130,939	16,367	961	501
PCR	175,530	21,941	2,419	1,733	175,524	21,941	2,416	1,731

Additionally, despite using Opti primers with an overhanging tail longer than the recommended length to account for potential truncation of sequencing reads due to transposase activity in the Rapid run, our results indicated that this was still insufficient to fully sequence the entire influenza genome segments and read coverage was still missing at the 3’- and 5’-ends. This, in turn, resulted in incomplete influenza genome assembly and significantly lower minimum coverage in the Rapid run when compared to the ligase-based Native and PCR runs.

### 3.3 Barcoded primers combined Native barcoding performance with Rapid barcoding preparation time

**Figure 4:**
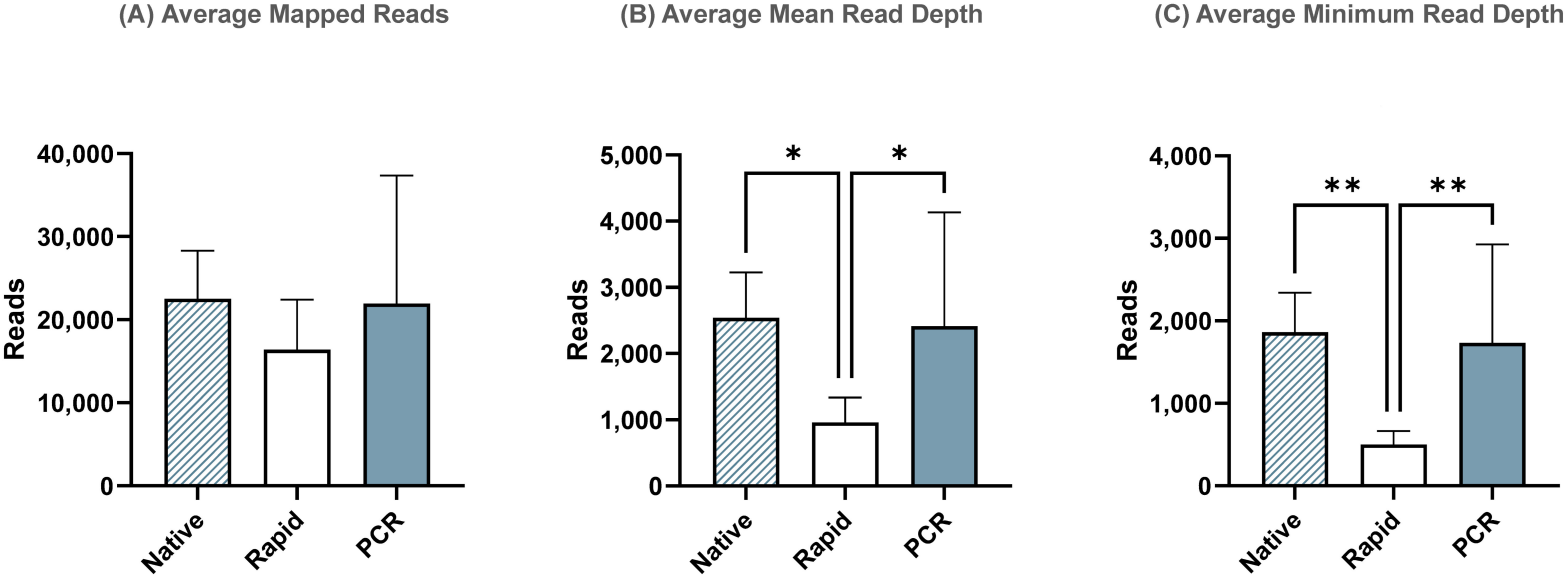
Sequencing summary for comparison of Native, Rapid, and PCR barcoding strategies’ performance with data mapped to reference sequences containing only coding regions to avoid potential bias from transposase cutting termini during Rapid-based barcode attachment. Average mapped **(A)**, mean read depth **(B)**, and minimum read depth **(C)** across all segments of eight different influenza A viruses. *P*-value is defined as follows: *p ≤ 0.05, **p ≤ 0.01.

To account for transposase-related termini truncation during rapid-based barcode attachment, we re-analyzed sequencing runs using shorter reference sequences containing only coding regions. While the average total number of influenza reads (**Figure 4A**) and mean read depth (**Figure 4B**) remained similar to those observed in the complete genome assembly, the minimum read depth significantly increased in the Rapid run for the coding-complete assembly (**Figure 4C**). However, it was still significantly lower compared to the ligase-based Native and PCR runs. This indicates that termini regions, which were not used in the coding-complete minimum read depth of the complete genome assembly.

**Figure 5:**
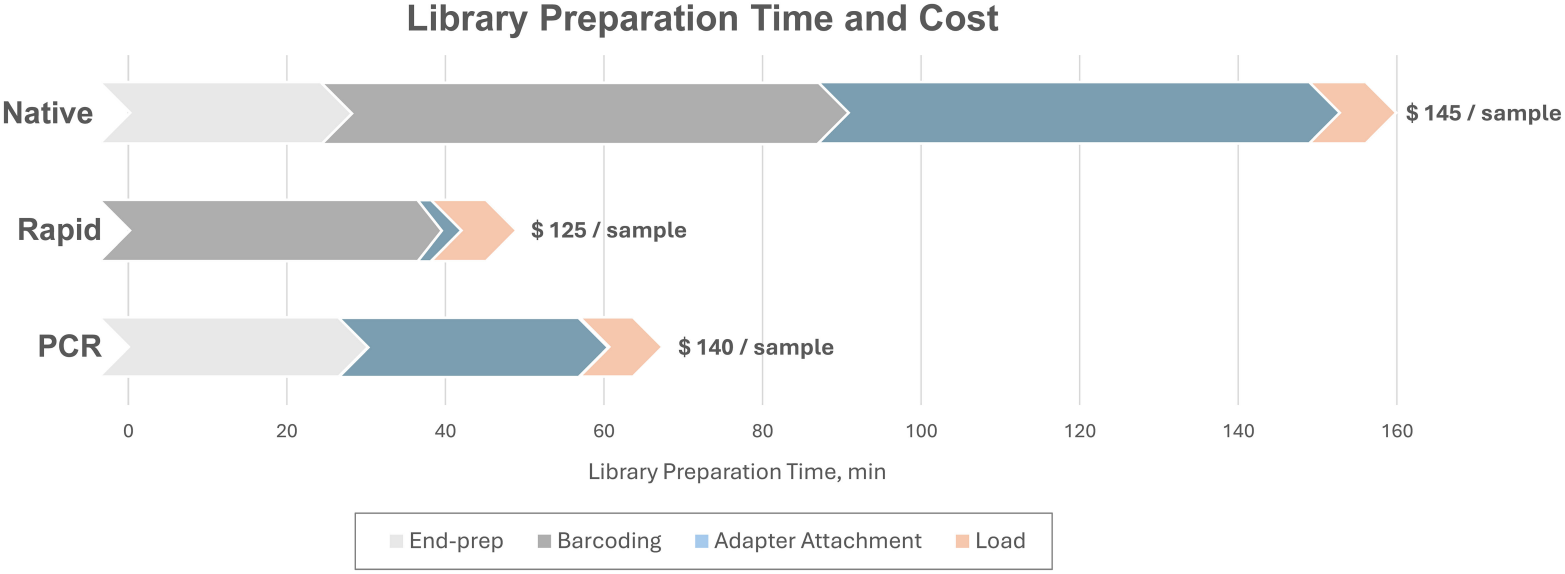
Comparison of time and cost of library preparation for Native, Rapid, and PCR sequencing runs on MinION flow cell. Library preparation time presented excludes RT-PCR amplification and amplicon purification. The cost presented excludes nucleic acid extraction, RT-PCR amplification, and flow cell. The current cost of R10.4.1 MinION flow cell ranges between $450 and $700 per flow cell depending on the purchased batch size, while the Flongle flow cell costs $810 per batch of 12 flow cells.

Overall, the PCR run using custom barcoded primers performed similarly to the Native Barcoding Kit, with no statistically significant differences in the average number of influenza reads, mean read depth, or minimum read depth across both complete genome and coding-complete genome assemblies. Notably, the PCR method also provided a substantial reduction in library preparation time, 2.3X faster than the Native Barcoding Kit, and was only 15 minutes longer than the Rapid Barcoding Kit (**Figure 5**). Considering that the custom barcoding primer approach delivers comparable performance to the Native Barcoding Kit, with similar preparation times to the Rapid kit and a reasonable cost (~$140 per sample for MinION sequencing or ~$70 per sample for Flongle sequencing). It presents an attractive alternative that combines the benefits of both methods, offering a balanced trade-off between speed, cost, and sequencing quality.

### 3.4 PCR method with barcoded primers validated on clinical samples

**Figure 6:**
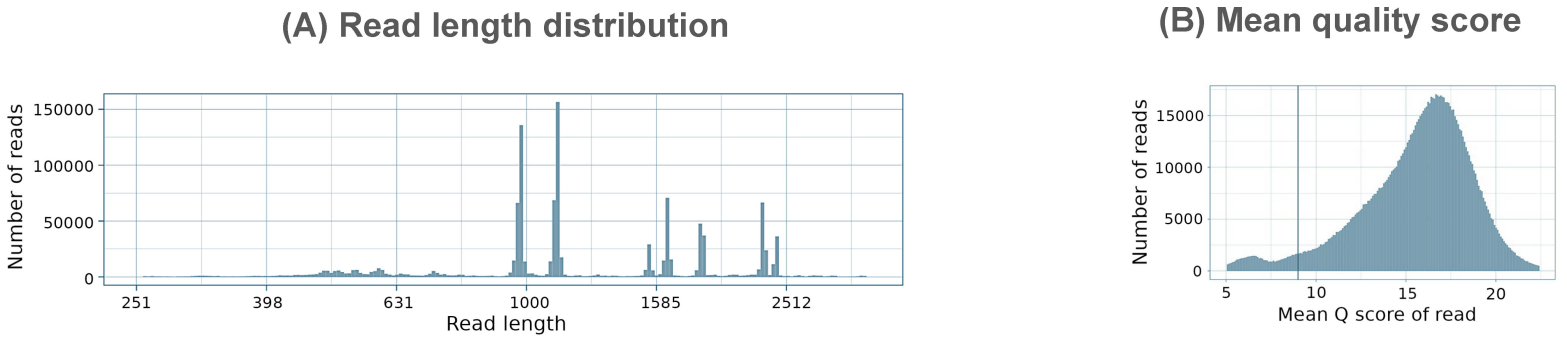
Summary of **(A)** read length distribution and **(B)** mean quality distributions in PCR sequencing run with clinical samples.

The PCR method utilizing barcoded primers was additionally tested on 12 clinical samples from different hosts and sample types. The RT-qPCR cycle threshold values for these samples ranged from 8.8 to 23.2, reflecting a different range of viral loads in tested samples (**Table 2**). Sequencing of 50 fmol of NGS library prepared with the PCR method for 6 hours generated 1,218,695 raw reads. Of these, 94.6% were successfully basecalled, with an average read length of 1344.4 bp and a mean quality score of 15.5 (**Figure 6**).

**Figure 7:**
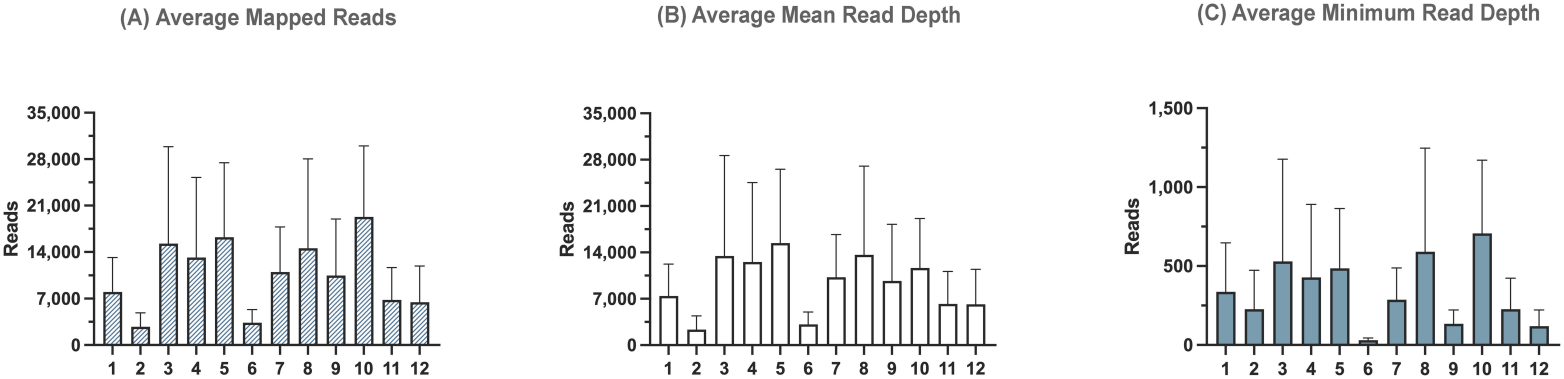
Sequencing summary for PCR barcoding strategies’ performance with clinical samples. Sequencing data was mapped to full-length reference genomes. Average mapped **(A)**, mean read depth **(B)**, and minimum read depth **(C)** across all segments of influenza A viruses from twelve different clinical samples.

A total of 92.3% of the basecalled reads were successfully demultiplexed. Reference mapping analysis revealed that 95.8% of the demultiplexed reads aligned to influenza sequences (**Table 5**). The number of influenza reads per sample ranged from 21,991 to 154,359, indicating successful amplification and sequencing across the different clinical sample types. The average number of mapped reads across influenza segments ranged from 2,749 to 19,295, with the average mean read depth ranging from 2,348 to 15,427 and the average minimum read depth between 31 and 706 reads (**Figure 7**). Consensus assembly revealed genome coverage ranging from 84.81% to 100%, with all samples showing complete genome coverage except for one OP sample, which had the highest Ct value of 23.2 among all tested samples. This sample exhibited the lowest genome breadth of coverage at 84.8%, primarily due to insufficient read coverage of the PA segment, while the remaining segments had full coverage.

**Table 5:** Summary statistics of influenza A genome assembly from clinical samples using PCR barcoding strategies for NGS library preparation.

Sample No.	Sample Type	Total Influenza Reads	Average Mapped Reads	Average Mean Read Depth	Average Min Read Depth	Mean Read Length, bp	Mean Quality	Genome Breadth of Coverage, %
1	Mammary gland	64,161	8,020	7,422	337	1082.5	14.9	100
2	Mammary gland	21,991	2,749	2,348	225	1187.8	15.0	100
3	Brain	122,160	15,270	13,461	529	1064.8	14.8	100
4	Muscle	105,254	13,157	12,563	429	1246.5	14.9	100
5	Muscle	129,936	16,242	15,427	485	1035	15.0	100
6	Heart	27,045	3,381	3,133	31	1462	15.0	100
7	Spleen	88,136	11,017	10,241	286	1469	15.0	100
8	Brain	116,582	14,573	13,647	591	1217.7	15.0	100
9	Heart	83,988	10,499	9,702	133	1236.6	15.1	100
10	OP ^1^	154,359	19,295	11,646	706	771.3	14.9	100
11	CL ^2^	54,476	6,810	6,215	227	1250.2	15.0	100
12	OP	51,576	6,447	6,171	119	1021	15.0	84.8

^1^Oropharyngeal swab sample; ^2^Cloacal swab sample.

## 4 Discussions

In this study, we optimized influenza A whole-genome on the nanopore sequencing platform by developing a custom barcoded primer strategy designed to provide both speed and high-quality data. Our results demonstrate that the custom barcoded primer strategy offers performance comparable to the Native Barcoding Kit, while significantly reducing library preparation time by 2.3X compared to the Native kit, and only 15 minutes longer than the Rapid Barcoding Kit. Because the custom barcode provides longer and more uniform reads than the rapid kit, it will likely require shorter sequencing runs to produce enough data for analysis, which could make it the fastest method of the three. Additionally, this method is highly versatile and can be applied to various types of samples, including egg-inoculated and clinical samples, and may prove effective with environmental and field samples, without requiring modifications to the sample preparation process. These findings present a promising solution for influenza surveillance, offering a balanced trade-off between sequencing quality, time efficiency, and cost-effectiveness.

In this study, we compared the custom barcoded primer method with the Native and Rapid barcoding kits in terms of sequencing yield, read quality, read depth, and sequencing output. Both the Native and PCR runs, utilizing ligation-based adapter attachment chemistry, yielded significantly higher numbers of raw reads compared to the Rapid run. This result is consistent with the known reduction in sequencing output associated with the Rapid Barcoding Kit. The decrease in sequencing output is likely due to the less effective rapid-based adapter attachment chemistry and the transposase-based barcoding approach used in the Rapid Barcoding Kit, which fragments amplicons during barcode attachment. While some reduction in reads with the Rapid kit was expected, our results demonstrated that this method produced a 1.2X decrease in total reads compared to the ligase-based methods, which could limit its utility in applications that require high read coverage or require longer sequencing runs to produce enough usable data.

The basecalling efficiency in all three methods was high, with over 80% of raw reads being successfully basecalled in all runs. The PCR run, in particular, achieved the highest percentage of basecalled reads (84.4%) and demonstrated the longest mean read length (1248.1 bp), closely followed by the Native run (81.0% and 1207.7 bp, respectively). Longer reads are crucial for improving genome assembly and accuracy. In contrast, the Rapid run generated shorter reads (730.6 bp), consistent with the fragmentation caused by transposase activity. While this increases speed, it reduces overall sequencing output and introduces challenges in genome assembly, particularly for isolates with recombination events. The mean quality scores were similar across all three methods and were above the minimum requirement of Q9, with scores ranging between 12.5 and 13.1. The distribution of reads across the influenza genome was more uniform in the Native and PCR runs, with reads corresponding to the length of the eight influenza genome segments. In contrast, the Rapid run exhibited a large number of shorter reads due to transposase fragmentation, leading to incomplete genome coverage, especially at the termini, which negatively impacted overall genome coverage.

The comparison of barcode attachment efficiency revealed a clear difference in demultiplexing performance. The Native run, which used ligation-based chemistry, achieved the highest percentage (97.2%) of demultiplexed reads, followed by the PCR method (92.0%). The Rapid run, in contrast, showed the lowest demultiplexing efficiency (87.3%) and the highest percentage of unclassified reads (12.6%). This lower efficiency in the Rapid run is likely due to the less effective barcode and adapter attachment associated with the rapid-based chemistry. Incorrect barcode assignments occurred in both the Native and Rapid runs, but the number of incorrectly barcoded reads was low in both cases. The PCR method, which used custom barcoded primers, showed no incorrect barcode assignments, demonstrating the effectiveness of the optimized Dorado demultiplexing settings for custom barcode arrangements. Currently, demultiplexing for custom barcodes is not supported directly within the MinKNOW software or the EPI2ME wf-basecalling workflow. As a result, it must be performed using a command-line interface with the standalone Dorado basecaller. The specific settings for the custom barcoded primers used in this study are provided in **Supplementary Table 1**. During the optimization of these settings, we specifically aimed to achieve no incorrect barcode assignments, which are often observed with the default demultiplexing settings for both the Native and Rapid barcoding kits. While lowering the settings for the custom barcoded primers would improve the demultiplexing rate, we prioritized accuracy over quantity. Additionally, we developed a Nextflow workflow that incorporates all necessary files and settings to streamline the demultiplexing process for custom barcodes, making it more accessible. The Nextflow workflow is available at https://github.com/Goraichuk/Dorado_FluA_Custom_Demultiplexing.

In terms of influenza genome assembly, the PCR method produced the highest percentage of reads mapped to reference influenza genome (99.97%) out of all demultiplexed reads, followed closely by the Native (98.8%) and Rapid (98.7%) runs. While the total number of reads mapped across the influenza genome was not significantly different between the three methods, the depth of coverage varied considerably. The PCR method demonstrated similar median and minimum read depths to the Native run, indicating its potential to provide high-quality data with reduced preparation time. In contrast, the Rapid run exhibited lower average mean and minimum depths of mapped reads, suggesting that the reduced read lengths and poorer coverage in the Rapid run resulted in incomplete genome assemblies and less reliable depth across the genome segments. This is particularly critical for SNP analysis and mutation detection, where high-quality and deep coverage are essential. It is important to note that, despite following ONT’s recommendations for Rapid barcoding method to add an extra 15-20 nt to primers to account for regions that will be trimmed during transposase activity, the primers used in our study included an additional 24 nt. However, despite the longer primers it still resulted in reduced read coverage at the influenza termini. Therefore, we recommend considering this when designing primers for amplification in cases where amplicons will be used for library preparation with the Rapid Barcoding Kit.

In addition to differences in read quality and genome coverage, the Native and Rapid kits also differ in cost. The main cost difference lies in the required third-party consumables. The Native library preparation requires Blunt/TA Repair Mix, Ultra II End repair/dA-tailing Module, and Quick Ligation Module. The Rapid library preparation, in contrast, does not require any of these consumables. The PCR method, using custom barcoded primers, eliminates the need for Blunt/TA Ligase Master Mix but still requires the Ultra II End repair/dA-tailing Module and Quick Ligation Module for the adapter ligation. As a result, Native barcoding incurs a higher cost per sample compared to the Rapid and PCR methods. In contrast, the Rapid method is more cost-effective due to its lower reagent requirements, making it a cheaper alternative despite its limitations in read quality and genome coverage. The PCR method presents an appealing alternative, combining the benefits of both approaches.

Additionally, the PCR method utilizing custom barcoded primers was successfully evaluated on 12 clinical samples, including those from bovine mammary gland tissues, cat brain tissues, and chicken brain, muscle, heart, spleen, OP, and CL swab samples. These samples, which varied in viral load, demonstrated the method’s versatility and robustness across different host species and sample types. All samples, except for one with the highest Ct value of 23.2, achieved 100% genome coverage. The sample with the highest Ct value exhibited reduced coverage due to missing reads for the PA segment. However, this did not affect the ability to identify the influenza subtype, as both the HA and NA segments were fully covered. While the results demonstrate the method’s effectiveness for reliable subtype identification even in samples with lower viral loads, further testing is needed to determine its limit of detection. When comparing PCR method on egg-grown virus isolates on Flongle flowcell and clinical samples on MinION flowcell, slightly higher basecalling efficiency and mean quality scores were observed in clinical samples. This can be attributed to the higher performance of the MinION flowcell, rather than differences in sample types. The demultiplexing rate remained consistent at 92.0% for Flongle and 92.3% for MinION, indicating that the efficiency of RT-PCR amplification with custom barcoded primers remained the same across clinical samples. The percentage of influenza reads was slightly lower in clinical samples (95.8%) compared to egg-grown isolates (99.97%), suggesting a marginal reduction in sequencing efficiency for clinical samples.

Overall, while both ONT barcoding kits offer solutions for different needs, with the Native kit providing higher yield but requiring longer library preparation time and higher cost, and the Rapid method being more time- and cost-efficient but lacking in read depth and genome breadth of coverage, our study demonstrates that the custom barcoded primer strategy offers a promising solution for influenza A nanopore sequencing. This method provides both high throughput and time efficiency. With a preparation time 2.3X faster than the Native Barcoding Kit and only 15 minutes longer than the Rapid kit, it strikes an ideal balance between the speed of the Rapid kit and the high-quality results of the Native kit.

## 5 Conclusion

In summary, our custom barcoded primer strategy for nanopore sequencing provides a balanced approach for influenza A sequencing, offering both speed and high-quality results. While the Rapid Barcoding Kit offers speed, it sacrifices sequencing yield and read depth, which are essential for comprehensive genomic analysis. The Native Barcoding Kit provides high-quality data but requires longer preparation times. The custom barcoded primer method offers a promising alternative that combines the advantages of both methods, reducing preparation time without compromising sequencing quality, making it a valuable tool for influenza A surveillance and outbreak management.

## Data Availability

The datasets presented in the study were deposited in the NCBI Sequence Read Archive under BioProject PRJNA1188165 (https://www.ncbi.nlm.nih.gov/bioproject/PRJNA1188165).
