# Supplementary material for: Custom barcoded primers for influenza A nanopore sequencing: enhanced performance with reduced preparation time
Source: Front Cell Infect Microbiol. 2025 Apr 15;15:1545032. doi: 10.3389/fcimb.2025.1545032 (PMC12037546; doi:10.3389/fcimb.2025.1545032)
Supplement: Supplementary file 1 [file DataSheet1.pdf]

Supplementary Table 1. Custom barcoded primer sequences and required settings for Dorado custom demultiplexing. Conserved Uni 12 and Uni 13 influenza termini highlighted in red.

Barcode	Primer Name	Primer Sequence 5'->3'
**BC01**	BC01-Rest-F1	AAGAAAGTTGTCGGTGTCTTTGTGAGCAAAAGCAGG
	BC01-Pol-F2	AAGAAAGTTGTCGGTGTCTTTGTGAGCGAAAGCAGG
	BC01-All-R	AAGAAAGTTGTCGGTGTCTTTGTGAGTAGAAACAAGG
**BC02**	BC02-Rest-F1	TCGATTCCGTTTGTAGTCGTCTGTAGCAAAAGCAGG
	BC02-Pol-F2	TCGATTCCGTTTGTAGTCGTCTGTAGCGAAAGCAGG
	BC02-All-R	TCGATTCCGTTTGTAGTCGTCTGTAGTAGAAACAAGG
**BC03**	BC03-Rest-F1	GAGTCTTGTGTCCCAGTTACCAGGAGCAAAAGCAGG
	BC03-Pol-F2	GAGTCTTGTGTCCCAGTTACCAGGAGCGAAAGCAGG
	BC03-All-R	GAGTCTTGTGTCCCAGTTACCAGGAGTAGAAACAAGG
**BC04**	BC04-Rest-F1	TTCGGATTCTATCGTGTTTCCCTAAGCAAAAGCAGG
	BC04-Pol-F2	TTCGGATTCTATCGTGTTTCCCTAAGCGAAAGCAGG
	BC04-All-R	TTCGGATTCTATCGTGTTTCCCTAAGTAGAAACAAGG
**BC05**	BC05-Rest-F1	CTTGTCCAGGGTTTGTGTAACCTTAGCAAAAGCAGG
	BC05-Pol-F2	CTTGTCCAGGGTTTGTGTAACCTTAGCGAAAGCAGG
	BC05-All-R	CTTGTCCAGGGTTTGTGTAACCTTAGTAGAAACAAGG
**BC06**	BC06-Rest-F1	TTCTCGCAAAGGCAGAAAGTAGTCAGCAAAAGCAGG
	BC06-Pol-F2	TTCTCGCAAAGGCAGAAAGTAGTCAGCGAAAGCAGG
	BC06-All-R	TTCTCGCAAAGGCAGAAAGTAGTCAGTAGAAACAAGG
**BC07**	BC07-Rest-F1	GTGTTACCGTGGGAATGAATCCTTAGCAAAAGCAGG
	BC07-Pol-F2	GTGTTACCGTGGGAATGAATCCTTAGCGAAAGCAGG
	BC07-All-R	GTGTTACCGTGGGAATGAATCCTTAGTAGAAACAAGG
**BC08**	BC08-Rest-F1	TTCAGGGAACAAACCAAGTTACGTAGCAAAAGCAGG
	BC08-Pol-F2	TTCAGGGAACAAACCAAGTTACGTAGCGAAAGCAGG
	BC08-All-R	TTCAGGGAACAAACCAAGTTACGTAGTAGAAACAAGG
**BC09**	BC09-Rest-F1	AACTAGGCACAGCGAGTCTTGGTTAGCAAAAGCAGG
	BC09-Pol-F2	AACTAGGCACAGCGAGTCTTGGTTAGCGAAAGCAGG
	BC09-All-R	AACTAGGCACAGCGAGTCTTGGTTAGTAGAAACAAGG
**BC10**	BC10-Rest-F1	AAGCGTTGAAACCTTTGTCCTCTCAGCAAAAGCAGG
	BC10-Pol-F2	AAGCGTTGAAACCTTTGTCCTCTCAGCGAAAGCAGG
	BC10-All-R	AAGCGTTGAAACCTTTGTCCTCTCAGTAGAAACAAGG
**BC11**	BC11-Rest-F1	GTTTCATCTATCGGAGGGAATGGAAGCAAAAGCAGG
	BC11-Pol-F2	GTTTCATCTATCGGAGGGAATGGAAGCGAAAGCAGG
	BC11-All-R	GTTTCATCTATCGGAGGGAATGGAAGTAGAAACAAGG
**BC12**	BC12-Rest-F1	CAGGTAGAAAGAAGCAGAATCGGAAGCAAAAGCAGG
	BC12-Pol-F2	CAGGTAGAAAGAAGCAGAATCGGAAGCGAAAGCAGG
	BC12-All-R	CAGGTAGAAAGAAGCAGAATCGGAAGTAGAAACAAGG
**BC13**	BC13-Rest-F1	AGAACGACTTCCATACTCGTGTGAAGCAAAAGCAGG
	BC13-Pol-F2	AGAACGACTTCCATACTCGTGTGAAGCGAAAGCAGG
	BC13-All-R	AGAACGACTTCCATACTCGTGTGAAGTAGAAACAAGG
**BC14**	BC14-Rest-F1	AACGAGTCTCTTGGGACCCATAGAAGCAAAAGCAGG
	BC14-Pol-F2	AACGAGTCTCTTGGGACCCATAGAAGCGAAAGCAGG
	BC14-All-R	AACGAGTCTCTTGGGACCCATAGAAGTAGAAACAAGG
**BC15**	BC15-Rest-F1	AGGTCTACCTCGCTAACACCACTGAGCAAAAGCAGG
	BC15-Pol-F2	AGGTCTACCTCGCTAACACCACTGAGCGAAAGCAGG
	BC15-All-R	AGGTCTACCTCGCTAACACCACTGAGTAGAAACAAGG
**BC16**	BC16-Rest-F1	CGTCAACTGACAGTGGTTCGTACTAGCAAAAGCAGG
	BC16-Pol-F2	CGTCAACTGACAGTGGTTCGTACTAGCGAAAGCAGG
	BC16-All-R	CGTCAACTGACAGTGGTTCGTACTAGTAGAAACAAGG
**BC17**	BC17-Rest-F1	ACCCTCCAGGAAAGTACCTCTGATAGCAAAAGCAGG
	BC17-Pol-F2	ACCCTCCAGGAAAGTACCTCTGATAGCGAAAGCAGG
	BC17-All-R	ACCCTCCAGGAAAGTACCTCTGATAGTAGAAACAAGG
**BC18**	BC18-Rest-F1	CCAAACCCAACAACCTAGATAGGCAGCAAAAGCAGG
	BC18-Pol-F2	CCAAACCCAACAACCTAGATAGGCAGCGAAAGCAGG
	BC18-All-R	CCAAACCCAACAACCTAGATAGGCAGTAGAAACAAGG
**BC19**	BC19-Rest-F1	GTTCCTCGTGCAGTGTCAAGAGATAGCAAAAGCAGG
	BC19-Pol-F2	GTTCCTCGTGCAGTGTCAAGAGATAGCGAAAGCAGG
	BC19-All-R	GTTCCTCGTGCAGTGTCAAGAGATAGTAGAAACAAGG
**BC20**	BC20-Rest-F1	TTGCGTCCTGTTACGAGAACTCATAGCAAAAGCAGG
	BC20-Pol-F2	TTGCGTCCTGTTACGAGAACTCATAGCGAAAGCAGG
	BC20-All-R	TTGCGTCCTGTTACGAGAACTCATAGTAGAAACAAGG
**BC21**	BC21-Rest-F1	GAGCCTCTCATTGTCCGTTCTCTAAGCAAAAGCAGG
	BC21-Pol-F2	GAGCCTCTCATTGTCCGTTCTCTAAGCGAAAGCAGG
	BC21-All-R	GAGCCTCTCATTGTCCGTTCTCTAAGTAGAAACAAGG
**BC22**	BC22-Rest-F1	ACCACTGCCATGTATCAAAGTACGAGCAAAAGCAGG
	BC22-Pol-F2	ACCACTGCCATGTATCAAAGTACGAGCGAAAGCAGG
	BC22-All-R	ACCACTGCCATGTATCAAAGTACGAGTAGAAACAAGG
**BC23**	BC23-Rest-F1	CTTACTACCCAGTGAACCTCCTCGAGCAAAAGCAGG
	BC23-Pol-F2	CTTACTACCCAGTGAACCTCCTCGAGCGAAAGCAGG
	BC23-All-R	CTTACTACCCAGTGAACCTCCTCGAGTAGAAACAAGG
**BC24**	BC24-Rest-F1	GCATAGTTCTGCATGATGGGTTAGAGCAAAAGCAGG
	BC24-Pol-F2	GCATAGTTCTGCATGATGGGTTAGAGCGAAAGCAGG
	BC24-All-R	GCATAGTTCTGCATGATGGGTTAGAGTAGAAACAAGG

Custom_barcode_arrs.toml	Custom_barcodes.fasta

```
[arrangement]
name = "BC"
kit = "BC"

mask1_front = ""
mask1_rear = "AGCRAAAGCAGG"
mask2_front = ""
mask2_rear = "CCTTGTTTCTACT"

# Barcode sequences
barcode1_pattern = "fwd%02i"
barcode2_pattern = "rev%02i"
first_index = 1
last_index = 24

## Scoring options
[scoring]
max_barcode_penalty = 10
barcode_end_proximity = 75
min_barcode_penalty_dist = 4
min_separation_only_dist = 5
flank_left_pad = 15
flank_right_pad = 15
front_barcode_window = 90
rear_barcode_window = 65
```

```
>fwd01
AAGAAAGTTGTCGGTGTCTTTGTG
>rev01
CACAAAGACACCGACAACTTTCTT
>fwd02
TCGATTCCGTTTGTAGTCGTCTGT
>rev02
ACAGACGACTACAAACGGAATCGA
>fwd03
GAGTCTTGTGTCCCAGTTACCAGG
>rev03
CCTGGTAACTGGGACACAAGACTC
>fwd04
TTCGGATTCTATCGTGTTTCCCTA
>rev04
TAGGGAAACACGATAGAATCCGAA
>fwd05
CTTGTCCAGGGTTTGTGTAACCTT
>rev05
AAGGTTACACAAACCCTGGACAAG
>fwd06
TTCTCGCAAAGGCAGAAAGTAGTC
>rev06
GACTACTTTCTGCCTTTGCGAGAA
>fwd07
GTGTTACCGTGGGAATGAATCCTT
>rev07
AAGGATTCATTCCCACGGTAACAC
>fwd08
TTCAGGGAACAAACCAAGTTACGT
>rev08
ACGTAACTTGGTTTGTTCCCTGAA
>fwd09
AACTAGGCACAGCGAGTCTTGGTT
>rev09
AACCAAGACTCGCTGTGCCTAGTT
>fwd10
AAGCGTTGAAACCTTTGTCCTCTC
>rev10
GAGAGGACAAAGGTTTCAACGCTT
>fwd11
GTTTCATCTATCGGAGGGAATGGA
>rev11
TCCATTCCCTCCGATAGATGAAAC
>fwd12
CAGGTAGAAAGAAGCAGAATCGGA
>rev12
TCCGATTCTGCTTCTTTCTACCTG
>fwd13
AGAACGACTTCCATACTCGTGTGA
>rev13
TCACACGAGTATGGAAGTCGTTCT
>fwd14
AACGAGTCTCTTGGGACCCATAGA
>rev14
TCTATGGGTCCCAAGAGACTCGTT
>fwd15
AGGTCTACCTCGCTAACACCACTG
>rev15
CAGTGGTGTTAGCGAGGTAGACCT
>fwd16
CGTCAACTGACAGTGGTTCGTACT
>rev16
AGTACGAACCACTGTCAGTTGACG
>fwd17
ACCCTCCAGGAAAGTACCTCTGAT
>rev17
ATCAGAGGTACTTTCCTGGAGGGT
>fwd18
CCAAACCCAACAACCTAGATAGGC
>rev18
GCCTATCTAGGTTGTTGGGTTTGG
>fwd19
GTTCCTCGTGCAGTGTCAAGAGAT
>rev19
ATCTCTTGACACTGCACGAGGAAC
>fwd20
TTGCGTCCTGTTACGAGAACTCAT
>rev20
ATGAGTTCTCGTAACAGGACGCAA
>fwd21
GAGCCTCTCATTGTCCGTTCTCTA
>rev21
TAGAGAACGGACAATGAGAGGCTC
>fwd22
ACCACTGCCATGTATCAAAGTACG
>rev22
CGTACTTTGATACATGGCAGTGGT
>fwd23
CTTACTACCCAGTGAACCTCCTCG
>rev23
CGAGGAGGTTCACTGGGTAGTAAG
>fwd24
GCATAGTTCTGCATGATGGGTTAG
>rev24
CTAACCCATCATGCAGAACTATGC
```

Dorado demux --emit-fastq --no-trim --output-dir /PATH/TO/OUTPUT_DIR --barcode-sequences /PATH/TO/custom_barcodes.fasta --barcode-arrangement /PATH/TO/custom_barcode_arrs.toml /PATH/TO/INPUT_DIR
