# Supplementary material for: Custom barcoded primers for influenza A nanopore sequencing: enhanced performance with reduced preparation time
Source: Front Cell Infect Microbiol. 2025 Apr 15;15:1545032. doi: 10.3389/fcimb.2025.1545032 (PMC12037546; doi:10.3389/fcimb.2025.1545032)
Supplement: Supplementary file 2 [file DataSheet2.pdf]

SUPPLEMENTAL TABLE

Data Availability

GISAID Identifier:	EPI_SET_250219sh
doi:	10.55876/gis8.250219sh

All genome sequences and associated metadata in this dataset are published in GISAID's EpiFlu database. To view the contributors of each individual sequence with details such as accession number, Virus name, Collection date, Originating Lab and Submitting Lab and the list of Authors, visit 10.55876/gis8.250219sh

Data Snapshot

- EPI_SET_250219sh is composed of 8 individual viruses;
- The collection dates range from 2015-04-18 to 2024-01-01;
- Data were collected in 1 countries and territories.
